# Targeted Regulation Of Cgrp Gene Expression

**DOI:** 10.1100/tsw.2001.438

**Published:** 2001-12-12

**Authors:** A. F. Russo, P. L. Durham

**Affiliations:** Department of Physiology and Biophysics, University of Iowa, Iowa City, IA 52242, USA

## Abstract

The neuropeptide calcitonin gene-related peptide (CGRP) is a potent regulator of cerebral vascular tone and contributes to neurogenic inflammation. Clinical studies have shown that CGRP levels are elevated during the painful phase of migraine headache, then restored to baseline by antimigraine 5-HT1 drugs. Conversely, CGRP is depleted in perivascular nerve terminals from patients who have suffered vasospasm following subarachnoid hemorrhage. We have investigated the mechanisms controlling CGRP expression in the trigeminal ganglia neurons, which provide virtually all of the CGRP innervation to the cerebral vasculature. We found that nerve depolarization, inflammatory compounds, and nitric oxide can increase CGRP synthesis and secretion. Using both adenoviral vectors and transfection approaches, we have shown that the increased synthesis is due to activation of a cell-specific MAP kinase-responsive enhancer upstream of the CGRP gene. Interestingly, the 5-HT1 migraine drugs are able to block this up-regulation by a mechanism that involves a very prolonged elevation of calcium. We have shown that the duration of the calcium signal is a key determinant for whether a MAP kinase responsive gene will be stimulated or repressed by calcium-activated pathways. This observation supports the importance of a finely tuned balance of calcium in the trigeminal neuron, which is intriguing in light of genetic evidence for calcium channel mutations in a rare form of inherited migraine. These studies suggest that modulation of MAP kinase control of the cell-specific CGRP gene enhancer may be a useful therapeutic strategy for neurovascular disorders.

